# Supplementary figures and images for: Interrupted Time Series Analysis of Pediatric Infectious Diseases and the Consumption of Antibiotics in an Atlantic European Region during the SARS-CoV-2 Pandemic
Source: Antibiotics (Basel). 2022 Feb 18;11(2):264. doi: 10.3390/antibiotics11020264 (PMC8868325; doi:10.3390/antibiotics11020264)

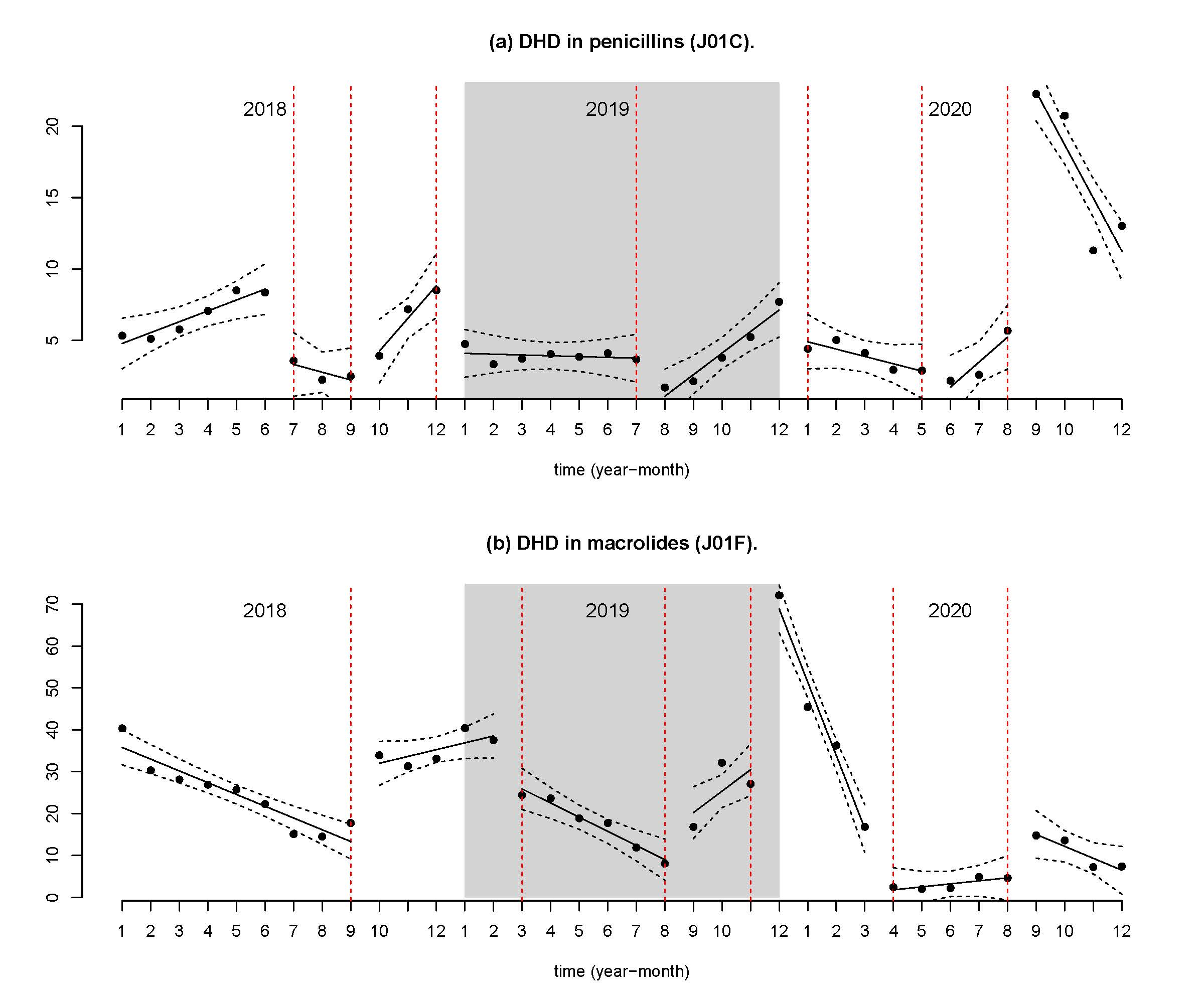

Supplement: Supplementary file 1 [file antibiotics-11-00264-s001.zip › Figure 2.rev.jpg]

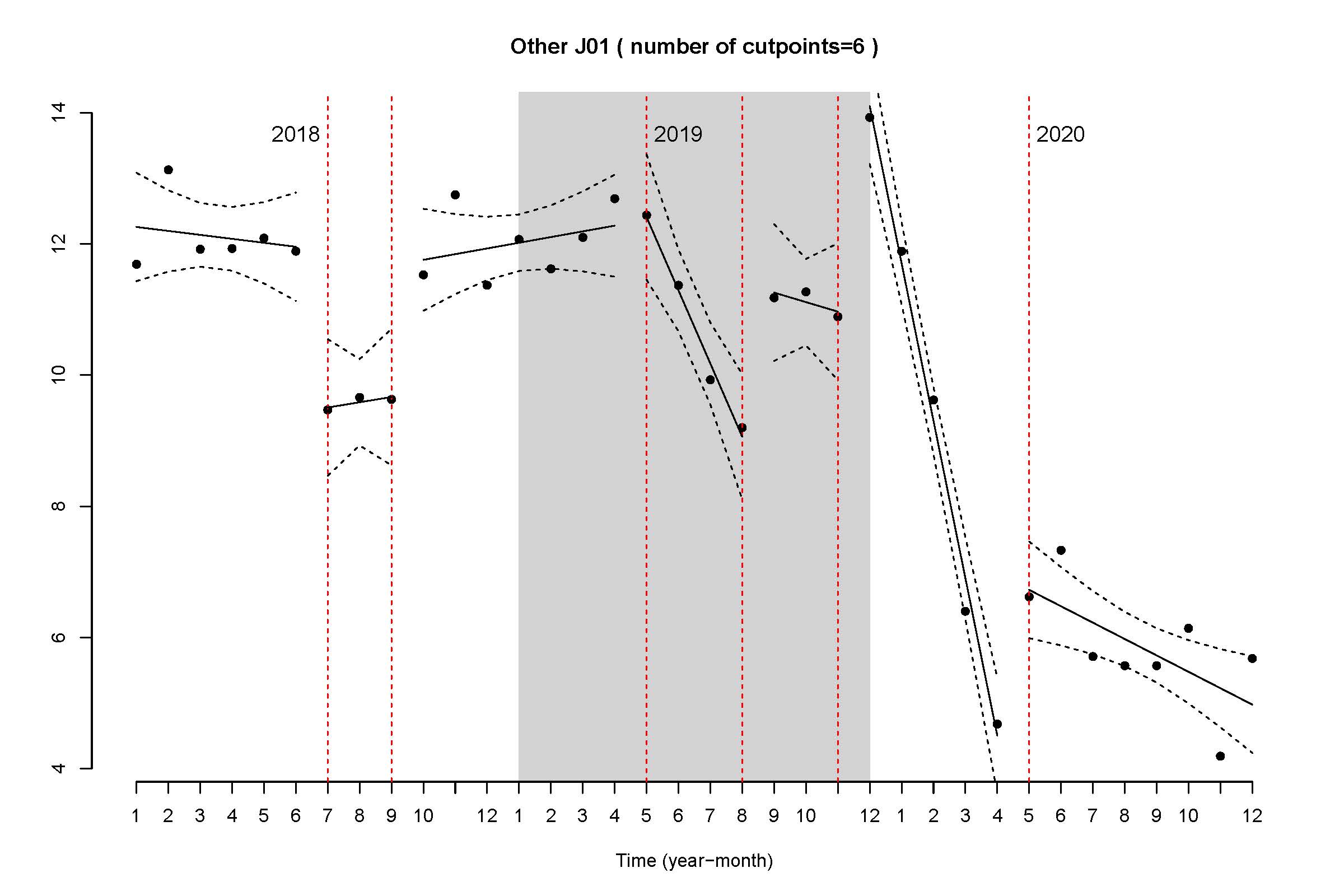

Supplement: Supplementary file 1 [file antibiotics-11-00264-s001.zip › Figure 3.jpg]

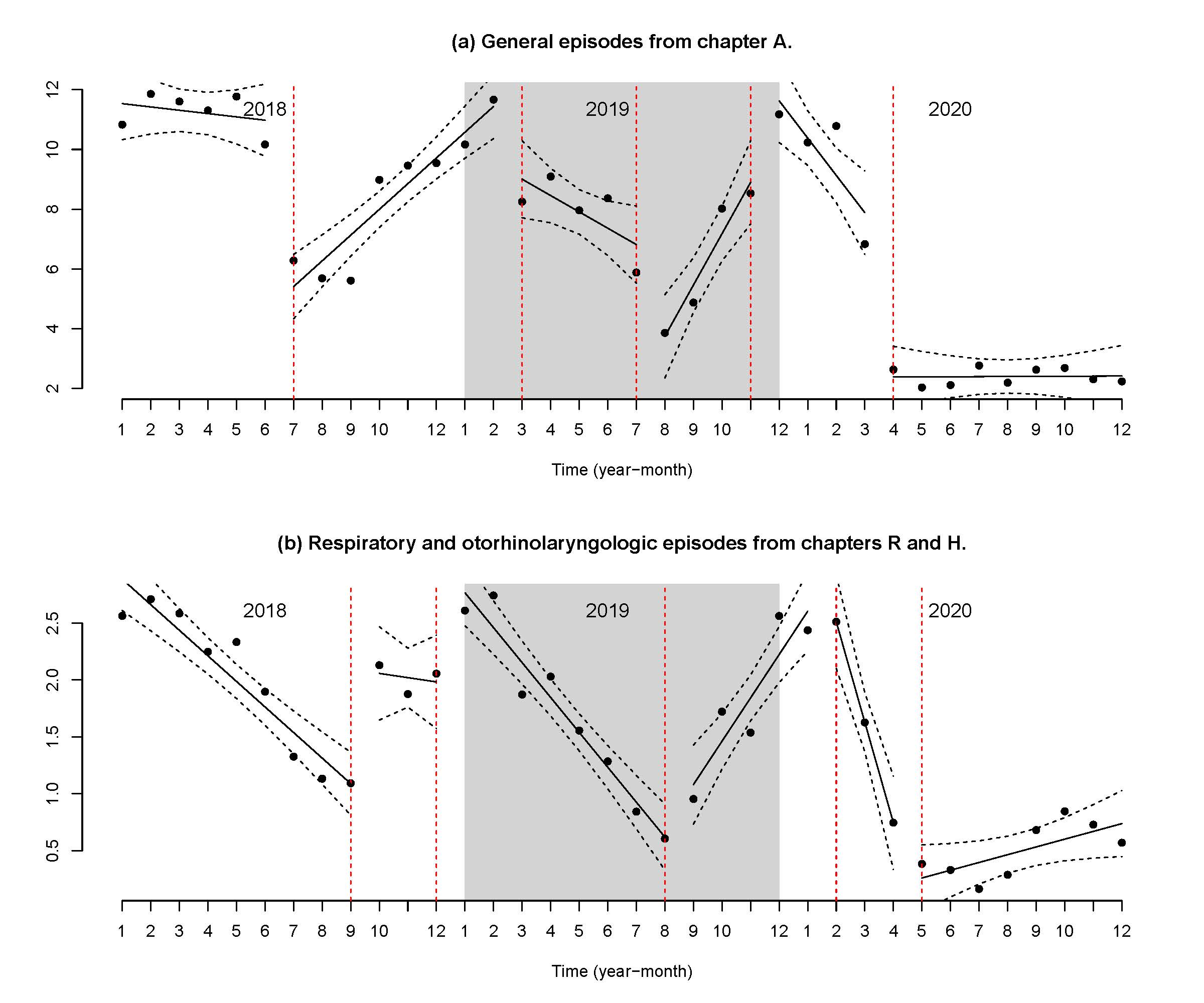

Supplement: Supplementary file 1 [file antibiotics-11-00264-s001.zip › Figure1.rev.jpg]

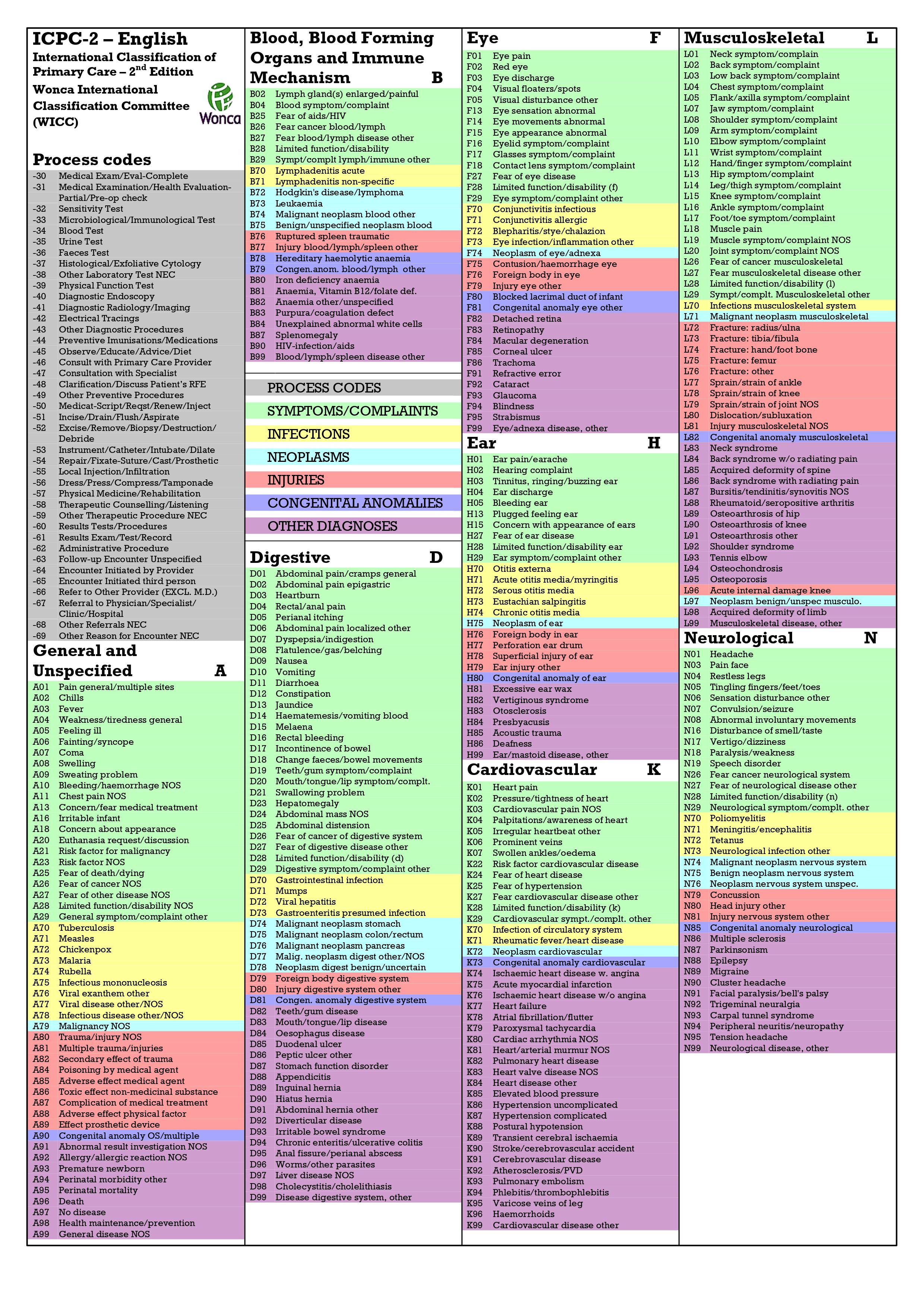

Supplement: Supplementary file 1 [file antibiotics-11-00264-s001.zip › File S1-1.jpg]

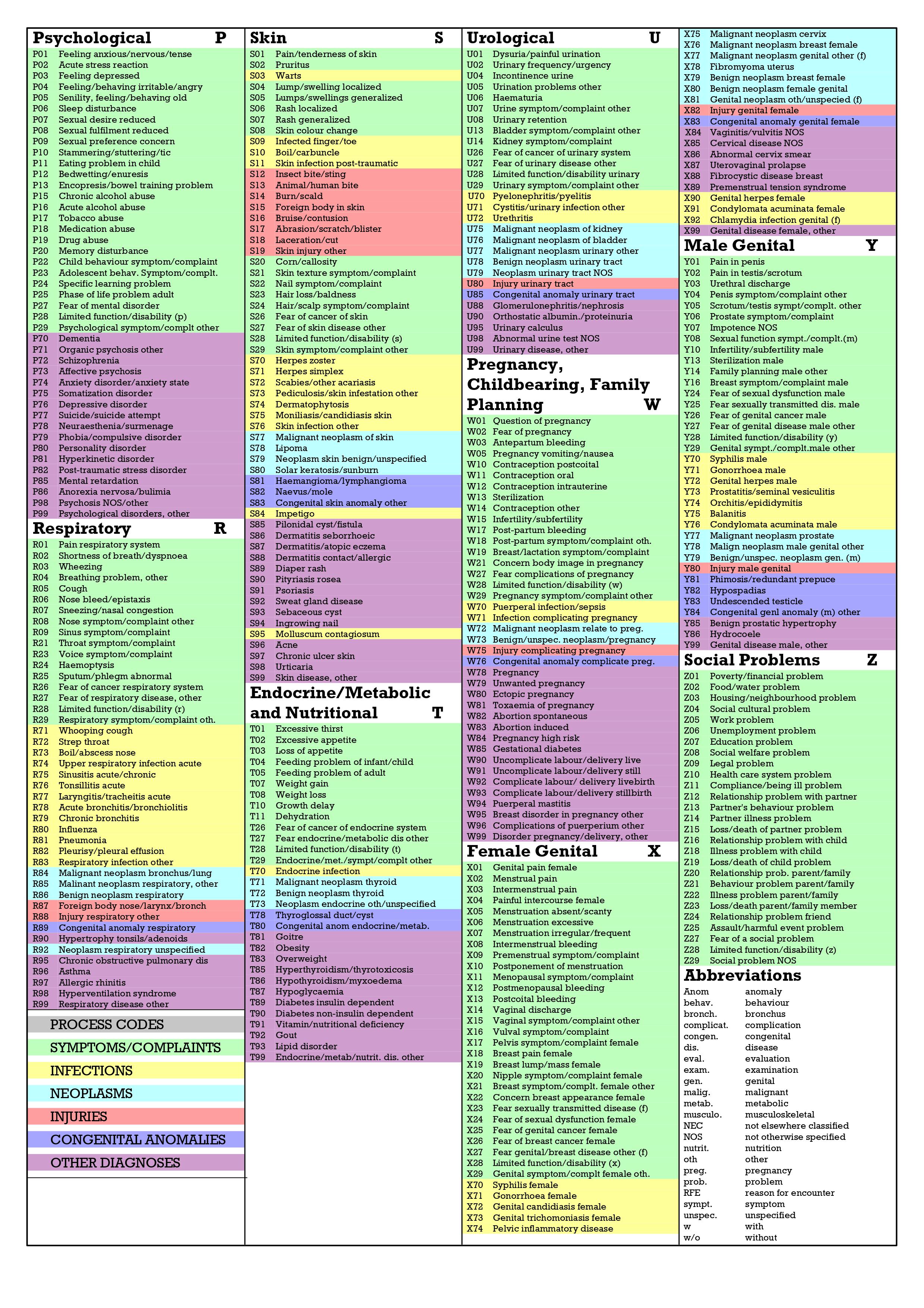

Supplement: Supplementary file 1 [file antibiotics-11-00264-s001.zip › File S1-2.jpg]
